# Impact Absorption Optimization in Rigid Polyurethane Foams Modified with Diethanolamine

**DOI:** 10.3390/polym18141741

**Published:** 2026-07-16

**Authors:** Tatiana Francisco, Fabio Oliveira, Rosana Moreira, Elcio Cruz de Oliveira, Diego Souza

**Affiliations:** 1 Laboratório de Ensaios de Produtos (LAENP), National Institute of Technology (INT), Rio de Janeiro 20081-312, RJ, Brazil; fabio.silva@int.gov.br (F.O.); rosana.moreira@int.gov.br (R.M.); 2 Institute of Macromolecules Professora Eloisa Mano (IMA), Federal University of Rio de Janeiro (UFRJ), Rio de Janeiro 21941-598, RJ, Brazil; diegosaboya@ima.ufrj.br; 3 Postgraduate Programme in Metrology, Pontifical Catholic University of Rio de Janeiro, Rio de Janeiro22451-900, RJ, Brazil; elciooliveira@puc-rio.br

**Keywords:** rigid polyurethane foam, impact attenuation, data envelopment analysis

## Abstract

Rigid polyurethane foams are used in impact-attenuation systems due to their tunable cellular structure and energy dissipation capacity. However, expanded polystyrene (EPS), commonly used for impact protection, presents limitations related to impact attenuation performance and limited design flexibility. This study evaluates the impact performance of rigid polyurethane foams modified with diethanolamine and assesses formulation efficiency using Data Envelopment Analysis (DEA). Rigid PU foam formulations containing 0–3 wt% DEOA were synthesized and characterized by impact testing, apparent density measurements, Scanning Electron Microscopy, Fourier Transform Infrared Spectroscopy, and Thermogravimetric Analysis/Derivative Thermogravimetry. DEA was applied to correlate diethanolamine content with impact absorption efficiency. Excessive crosslinking and reduced energy dissipation were observed above 2 wt%, while concentrations below 0.5 wt% resulted in poorly structured foams. The formulation containing 1 wt% DEOA was identified as the most efficient among the investigated formulations, exhibiting the best overall performance, reducing transmitted peak acceleration by 13.8% compared with neat PU foam, while exhibiting an approximately 48% increase in apparent density, more complete consumption of NCO groups, a more uniform cellular structure, and only modest changes in thermal degradation behavior. These findings indicate that the improved impact performance is associated with the combined effects of increased apparent density, modified cellular morphology, and changes in the polyurethane network promoted by DEOA, underscore the promise of diethanolamine-modified rigid polyurethane (PU) foams for protective applications.

## 1. Introduction

Polyurethanes (PU) constitute a versatile class of polymers widely used in impact-attenuation systems due to their tunable chemical structures and ability to form foams with varying densities, elasticities, and energy-dissipation levels [1,2]. Their highly controllable cellular morphology enables the tailoring of mechanical, thermal, and dynamic properties to a wide range of industrial applications, including packaging, mattresses, thermal insulation, automotive components, footwear, and other applications requiring controlled energy dissipation [1,2,3]. In such contexts, impact performance is a critical attribute, especially for helmets, knee pads, sports protectors, and passive safety systems, where the ability to attenuate accelerations and distribute mechanical energy determines product effectiveness and user safety.

Polyurethane foam has attracted attention for impact-absorbing systems and may offer formulation versatility compared with traditional materials such as expanded polystyrene (EPS) [4]. Although EPS is widely used due to its lightweight and good energy dissipation capacity, it presents important limitations, including low resistance to repeated impacts, brittle behavior at low temperatures, and recycling challenges arising from its thermoplastic nature and highly expanded structure [5,6].

In contrast, PU foams offer advantages such as higher toughness, the ability to tailor the microstructure through formulation, and greater potential for reuse and integration into sustainable material pathways [7,8]. Additionally, PU can incorporate natural fillers, reactive additives, and modifying agents that enhance performance under complex mechanical loading conditions [7,8].

Among additives used in PU formulations, diethanolamine plays a central role in modulating the structure of the final polymer. This compound acts simultaneously as a chain extender and as a nucleophilic reactant toward isocyanate (–NCO) groups, directly influencing the balance between gelling and blowing reactions [9,10,11].

Its presence can significantly alter the crosslink density, cell size, and regularity of the foam, the material stiffness, and, consequently, its impact attenuation properties. Excessive concentrations of diethanolamine may increase rigidity and reduce the capacity for plastic deformation, whereas insufficient levels can lead to less structured foams with poorer cell uniformity and inconsistent mechanical properties. Therefore, determining the optimal diethanolamine content is essential to ensure foams with stable and well-balanced performance [12].

Diethanolamine allows controlled expansion of carbon dioxide (CO_2_) during the polymerization reaction, promoting more uniform and gradual bubble formation and thus contributing to a more homogeneous foam structure. However, if the effectiveness of this addition has not been properly evaluated, the use of this compound may result in excessive or insufficient expansion, compromising foam quality [13].

According to previous results, diethanolamine can lead to the formation of foams with more uniform and well-defined cells; however, it can also interact with isocyanates and polyols, potentially causing undesirable effects in the polymerization process [14].

From an industrial perspective, diethanolamine may influence thermal properties and impact attenuation behavior, depending on formulation and processing conditions. Nevertheless, if not properly controlled, its use may increase production costs and introduce additional complexity into the formulation process [15,16].

Determining this optimal concentration, however, is not trivial, since the effect of diethanolamine depends on correlated variables—such as density, reaction time, cell uniformity, thermal behavior, and compressive strength. Traditional direct comparison methods may fail to capture nonlinear relationships or efficiency thresholds. In this context, Data Envelopment Analysis (DEA), particularly the FDH (Free Disposal Hull) and VRS (Variable Returns to Scale) approaches emerge as a robust tool for evaluating technical efficiency among different formulations DEA enables the simultaneous evaluation of formulation inputs, such as diethanolamine content, and performance outputs, including impact attenuation, dissipated energy, and transmitted peak acceleration, thereby allowing the identification of efficient and inefficient formulations. In this way, it mathematically identifies which formulations are efficient and which exhibit waste or suboptimal performance. In the case of PU foams, DEA helps determine the point at which increasing diethanolamine content no longer contributes to a real gain in mechanical performance [17,18].

In addition, the physicochemical characterization of the foams is essential to understanding the mechanisms underlying the results observed by DEA. Techniques such as Scanning Electron Microscopy (SEM), Infrared Spectroscopy (FTIR), Thermogravimetric Analysis (TGA), and Derivative Thermogravimetric Analysis (DTG) provide structural, chemical, and thermal evidence that help correlate formulation, morphology, and mechanical performance. Comparing foams with and without diethanolamine enables isolation of the specific contribution of this additive to the material structure, providing scientific support for formulation optimization [19,20,21,22].

Because apparent density strongly influences the mechanical response of cellular polymers, impact attenuation results must be interpreted together with density-related effects. Previous studies on polyurethane foams have shown that density affects compressive strength, stiffness, densification behavior, and energy absorption capacity. In this study, the relevance of DEOA incorporation is therefore interpreted as the combined result of increased apparent density, improved cellular organization, and modification of the polyurethane network. Thus, the reduction in transmitted peak acceleration remains meaningful because it was achieved in a formulation that also showed improved structural organization and was identified as efficient by the DEA approach, rather than simply representing a denser foam.

Considering the growing need for materials with improved mechanical performance and greater energy dissipation capacity in safety-related applications, especially in personal protective equipment, the development of rigid polyurethane foams with optimized microstructures for impact attenuation remains an important research challenge.

Although DEOA has been extensively studied in flexible polyurethane foams, particularly regarding morphology and structure–property relationships, its effect on impact attenuation in rigid polyurethane foams remains poorly understood. Furthermore, no previous studies were found combining impact attenuation analysis, DEA-based optimization, and physicochemical characterization to evaluate DEOA-modified rigid polyurethane foams.

In this context, modifying the formulation with diethanolamine (DEOA) emerges as a promising strategy to simultaneously adjust reactivity, cellular morphology, and crosslink density of the material. This study evaluates the influence of DEOA not only on mechanical behavior, but also on the chemical, thermal, and morphological characteristics of rigid polyurethane foams. In addition, the determination of the most efficient concentration of this additive using Data Envelopment Analysis (DEA) provides a complementary approach for optimizing formulation performance.

The present results suggest that DEOA content is associated with changes in transmitted peak acceleration, likely through combined effects on cellular morphology, chemical structure, and possibly density-related structural variations.

## 2. Materials and Methods

This section describes the raw materials, the foam processing, the boundary conditions for SEM, FTIR, TGA/DTG, the impact test, and DEA.

### 2.1. Raw Materials

The foam was produced from a commercial two-component rigid polyurethane system (polyol + isocyanate) supplied by AVIPOL^®^ (Avipol Comercial Ltda., Santo André, SP, Brazil). Component A (polyester polyol) contained surfactants, catalysts, and residual water as a chemical blowing agent. Component B consisted of toluene diisocyanate (TDI). Diethanolamine (analytical grade) was used as a reactive additive to modify the formulation. Samples were prepared with different DEOA concentrations (0%, 0.5%, 1%, 1.5%, 2%, 2.5% and 3% by weight relative to the polyol).

### 2.2. Foam Processing

**Figure 1:**
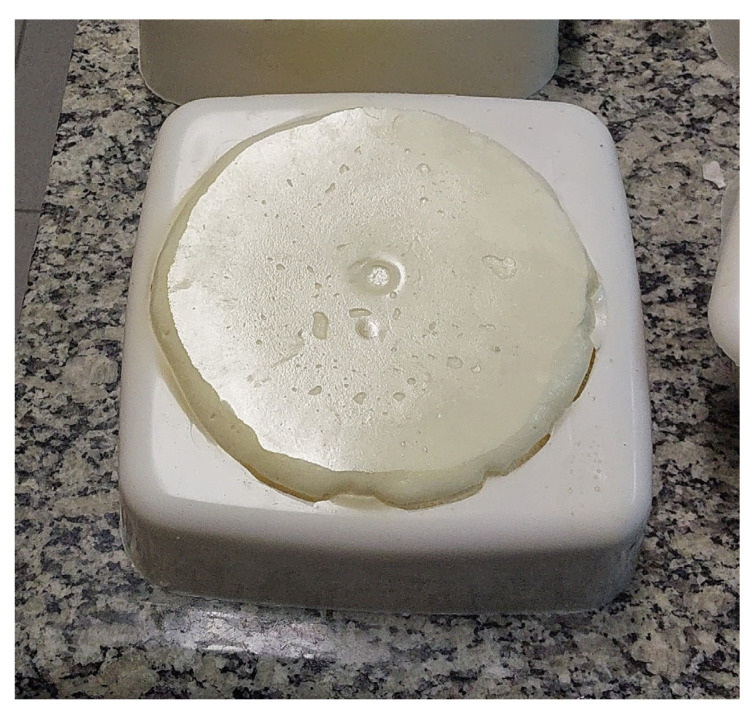
Polyurethane foam molding.

The polyol and isocyanate components were weighed in a 1:1 stoichiometric ratio. Diethanolamine (DEOA) was incorporated into the polyol phase immediately before processing, followed by vigorous mechanical mixing for 30 s to ensure homogeneous dispersion. The reactive mixture was then poured into a square mold (14 cm × 14 cm) containing a hollow cylindrical insert with an internal diameter of 13 cm and a height of 3.5 cm (Figure 1), producing cylindrical rigid polyurethane foam specimens with controlled dimensions. After expansion and curing, the foams were removed from the mold and conditioned at room temperature for 48 h prior to characterization and impact testing.

All foam specimens were produced under identical processing conditions and mold geometry.

### 2.3. Scanning Electron Microscopy (SEM)

The samples were analyzed without fracture preparation, using small sections of the foam directly mounted on metallic stubs. To ensure charge dissipation during analysis, the surfaces were previously coated with an ultrathin silver film obtained by sputtering. The micrographs were acquired using an electron microscope operated at an accelerating voltage (HV) of 2.00 kV, with an Everhart–Thornley detector (ETD) in backscattered detection mode, and a beam current of 86 pA.

The acquired SEM images were further processed and quantitatively analyzed using ImageJ^®^ software (version 1.54g, National Institutes of Health, USA). The images were calibrated using the scale bar provided in each micrograph, and the pore regions were segmented from the polymer matrix using the threshold function. The default thresholding method was applied for the quantitative analysis of the SEM images, except for the lower mold surface of the PU-DEOA foam, for which the Intermodes thresholding method was used due to differences in contrast between the cellular structure and the polymer matrix. Particle analysis was performed using the Analyze Particles tool, considering a size range of 10–∞ µm^2^ for both upper and lower surfaces to exclude small artifacts and image noise while maintaining representative cellular structures. The evaluated parameters included the number of pores, total analyzed area, pore area fraction (porosity), circularity, and other geometrical descriptors obtained from ImageJ^®^. All images within each comparison group were processed using the same analysis criteria to ensure consistency and reliability of the quantitative results.

### 2.4. Attenuated Total Reflectance Infrared Spectroscopy (ATR-FTIR)

The spectra were obtained in the range of 4000 to 400 cm^−1^, using 128 scans and a resolution of 4 cm^−1^, with a Bruker INVENIO-R spectrophotometer, to evaluate NCO conversion, the formation of urethane groups, and chemical changes associated with the presence of diethanolamine.

### 2.5. Thermogravimetric Analysis (TGA)

The curves were obtained from 30 to 900 °C under a nitrogen atmosphere, at a heating rate of 10 °C/min, to evaluate thermal stability, residual mass, and degradation profiles.

### 2.6. Impact Test

The impact tests were conducted to evaluate the ability of polyurethane foams, with and without the addition of diethanolamine, to dissipate mechanical energy. These tests were performed under controlled conditions. The methodology was adapted from the impact test described in the Brazilian Standard ABNT NBR 7471:2015 [23]. Testing was carried out at the Product Testing Laboratory (LAENP), located at the Brazilian National Institute of Technology (INT). This laboratory is accredited according to the Brazilian Standard ABNT NBR ISO/IEC 17025 [24], ensuring traceability, repeatability, and comparability of the results.

**Figure 2:**
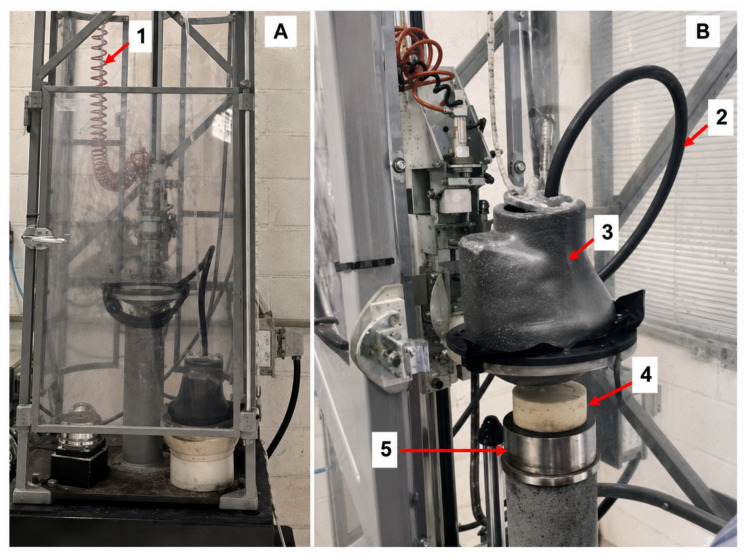
Drop tower apparatus used for impact attenuation testing. (**A**) General view of the impact testing system (1) coiled electrical cable. (**B**) Detailed view of the impact assembly showing the accelerometer cable (2), helmet shell (3), rigid polyurethane foam specimen (4), and support base (5).

The tests were performed using a free-fall impact testing machine, model MAU-1006 (AD Engineering^®^), equipped with a guided vertical tower, an electromagnetic release system, and an instrumented base with a high-sensitivity triaxial accelerometer (Figure 2). The data acquisition system was configured to record acceleration signals at a minimum sampling frequency of 20 kHz, preserving the resolution required for analyzing peak acceleration and the time history of the impact event.

Each foam specimen was positioned on a rigid base and impacted by a standard test head with a mass of 5.0 kg (Figure 2), released from an adjusted height of 50 cm to generate an approximate impact velocity of 3.1 m/s at the moment of contact.

Impact performance was evaluated based on the peak transmitted acceleration (g) recorded during impact. In the present study, g was not used as a unit of absorbed energy itself, but as the unit of the experimentally measured response adopted to assess impact attenuation performance. Lower acceleration values indicate greater impact attenuation and improved energy dissipation by the foam structure. Since all specimens were tested under identical impact conditions and geometry, transmitted peak acceleration was used as the primary performance metric for comparing formulations. For each formulation, (n = 10) specimens were tested, and the results are reported as mean ± standard deviation. The percentage reduction in transmitted peak acceleration was calculated relative to neat PU foam using the mean acceleration values obtained from the impact tests.

### 2.7. Apparent Density Measurement

The apparent density of the polyurethane foams was determined based on ASTM D1622 [25] for rigid cellular plastics. Cubic specimens with nominal dimensions of 3 × 3 × 3 cm were prepared from different regions of the foam samples to account for possible density variations along the structure. Prior to testing, all specimens were conditioned at room temperature (23 ± 2 °C) for 48 h to ensure moisture and thermal stabilization.

**Figure 3:**
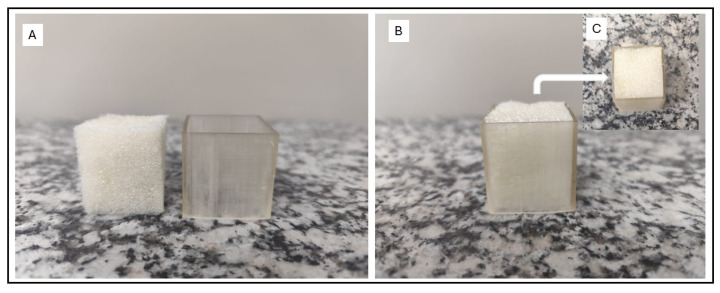
Preparation of standardized polyurethane foam specimens for apparent density measurements. (**A**) Polyurethane foam block and custom cutting mold manufactured by 3D printing. (**B**) Insertion of the foam into the mold for specimen shaping. (**C**) Final cubic specimen (3 × 3 × 3 cm) after trimming, ready for testing based on ASTM D1622.

The specimens were cut manually using a custom mold manufactured by additive manufacturing (3D printing) using a Creality Halot-L resin printer. The mold was fabricated using 3D Lab Standard resin, allowing reproducible cutting of the foam samples into standardized geometries. After cutting, the specimens were carefully trimmed to ensure dimensional uniformity as shown in Figure 3.

The mass of each specimen was measured using an analytical balance with a precision of 0.001 g. A total of 10 specimens were evaluated for each formulation (neat PU and PU–DEOA), and the results are reported as mean ± standard deviation.

The apparent density (ρ) was calculated according to Equation (1):

(1) $$\rho =\frac{m}{V}$$

where ρ is the apparent density (g·cm^−3^), *m* is the mass of the specimen (g), and *V* is the volume of the specimen (cm^3^).

All measurements were performed under controlled laboratory conditions (23 ± 2 °C).

## 3. Results and Discussion

After calibrating all equipment and performing the experimental procedures, the results are provided in four sections.

### 3.1. Data Envelopment Analysis: Efficiency and Optimal Diethanolamine Concentration

**Figure 4:**
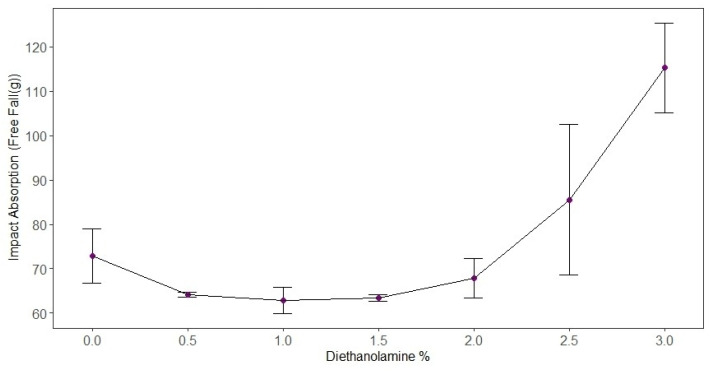
Transmitted peak acceleration of polyurethane foams containing different DEOA contents.

For each formulation, ten specimens (n = 10) were tested. The results from the impact tests were processed using R software version 4.3.2 (R Foundation for Statistical Computing, Vienna, Austria) (Figure 4). Although the literature has already suggested that concentrations above 2.0 wt% are not recommended for foam applications [26,27,28,29,30], this study investigated higher percentages, as shown in Figure 4. This decision was made because previous studies did not evaluate impact absorption, leaving a gap that is addressed in the present research.

The physical and mechanical performance of the synthesized rigid polyurethane (PU) foams is deeply governed by a complex trade-off between chemical crosslinking, cell morphology, and apparent density distribution. Diethanolamine (DEOA) plays a crucial role as a crosslinking agent, directly affecting the kinetic balance between the blowing (gas expansion) and gelling (polymerization) reactions.

When incorporated at the optimal concentration of 1 wt%, DEOA moderately increases the viscosity of the reacting medium during the initial expansion stage. This timely increase in viscosity enhances gas retention within the cellular matrix, preventing premature gas escape and leading to a highly homogeneous structure dominated by well-defined closed cells. Morphologically, this uniform microstructure may reduce is associated with severe density gradients across the mold, which translates into a more isotropic material capable of progressive deformation. Consequently, this optimized cellular framework ikely contributes to the maximum energy dissipation efficiency observed, yielding a 13.8% reduction in transmitted peak acceleration.

Conversely, incorporating higher concentrations of DEOA (≥2 wt%) triggers a severe imbalance in the reaction kinetics. At these elevated thresholds, excessive crosslinking causes the gelling reaction to occur too rapidly, leading to premature immobilization of the polyurethane matrix. As the blowing gas continues to expand against a prematurely rigidified and stiffened polymer network, localized internal pressures build up, promoting cell wall rupture and subsequent coalescence. As evidenced by Scanning Electron Microscopy (SEM) analysis, this phenomenon results in a highly heterogeneous structure characterized by larger, collapsed, and irregular cell cavities. This severe structural degradation destroys the foam’s ability to undergo uniform progressive collapse under dynamic loading, ultimately compromising its impact absorption capacity.

From a physicochemical standpoint, the increase in diethanolamine content accelerates the formation of urea and urethane linkages due to the greater availability of hydroxyl and amine groups. At higher concentrations, these reactions occur more rapidly and less uniformly, altering the balance between blowing and gelling reactions. This imbalance may also affect local structural organization and contribute to heterogeneity within the foam. As a result, even when macroscopic density appears similar, the internal cellular structure becomes more irregular.

The results suggest that higher DEOA concentrations may favor earlier network formation and increased structural rigidity, leading to premature cell stabilization and restricted expansion. This phenomenon results in irregular, partially collapsed cells or thicker cell walls, increasing cellular anisotropy. Consequently, the foam exhibits heterogeneous mechanical behavior, with rigid domains coexisting with less structured regions. This structural variability explains the greater dispersion observed in the impact results at higher concentrations.

Figure 4 shows the average transmitted peak acceleration values and the corresponding standard deviations (error bars) for each diethanolamine concentration. Concentrations of 0.5 wt%, 1.0 wt%, and 1.5 wt% present the lowest peak acceleration values, ranging from 60 g to 70 g.

To quantitatively assess the effect of DEOA addition on impact attenuation performance, the percentage reduction in transmitted peak acceleration was calculated relative to the neat PU foam according to Equation (2), where APU represents the mean transmitted peak acceleration of the unmodified foam and ADEOA corresponds to the mean transmitted peak acceleration of the DEOA-modified formulation. This approach allows a direct comparison of the effectiveness of each formulation in reducing impact-induced acceleration and provides a quantitative measure of the improvement achieved by DEOA incorporation.

(2) $$Reduction\;\left(\%\right)=\frac{\;A_{\left\{PU\right\}}-A_{\left\{DEOA\right\}}}{A_{\left\{PU\right\}}}\;\;$$

where $A_{\left\{PU\right\}}\;$ is the mean transmitted peak acceleration of the neat polyurethane foam and ${A\;}_{(DEOA)\;}\;$ is the mean transmitted peak acceleration of the foam containing DEOA.

Table 1 summarizes the mean transmitted peak acceleration values obtained for the polyurethane foams containing different DEOA concentrations. The formulation containing 1.0 wt % DEOA exhibited the lowest transmitted peak acceleration (62.79 g), indicating the best impact attenuation performance among all evaluated formulations. In comparison, neat PU foam presented an average transmitted peak acceleration of 72.88 g. This corresponds to a reduction of 13.84%, demonstrating the beneficial effect of DEOA addition on impact performance.

The increased variability observed at certain concentrations is attributed to microstructural heterogeneity arising from localized differences in reaction kinetics and crosslink density. Even under identical processing conditions, small variations in cellular morphology – such as cell size distribution and cell wall thickness – can influence impact response, resulting in dispersion of the measured acceleration values. Such behavior is inherent to rigid polyurethane foams and has been widely reported in the literature [12,23,28].

To determine which of these concentrations is most efficient, balancing diethanolamine content and impact attenuation performance, the analysis focused on identifying the optimal point that minimizes input and maximizes output.

For this purpose, two DEA (Data Envelopment Analysis) models were applied: (i) Free Disposal Hull (FDH); and (ii) Variable Returns to Scale (VRS).

The FDH model assumes that inefficient units can eliminate any amount of input without reducing output. In this case, efficient units are those in which the diethanolamine concentration is minimized without negatively affecting impact attenuation performance. It is important to note that FDH evaluates efficiency based on the dataset by identifying units closest to the efficiency frontier and using resources most effectively.

The VRS model, on the other hand, evaluates the efficiency of each unit relative to the efficiency frontier while allowing for variable returns to scale. This model is also input-oriented.

**Figure 5:**
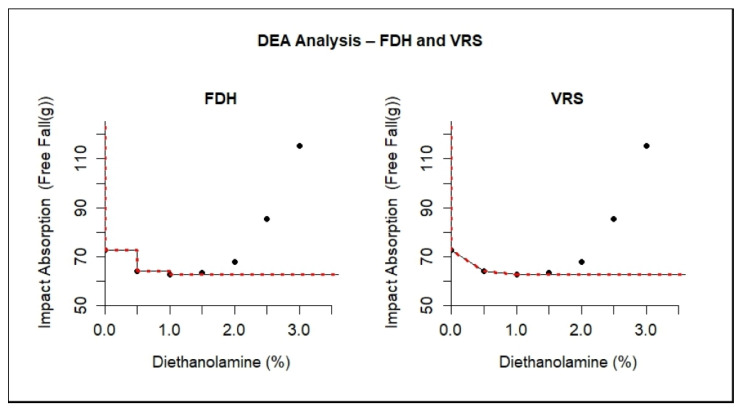
Data Envelopment Analysis (DEA) results for polyurethane foams with different DEOA contents. The dashed red line represents the efficiency of the frontier. Points on the frontier are considered efficient, while those above indicate inefficient formulations.

Figure 5 presents the DEA results, where the dashed red line represents the efficiency frontier, and each point corresponds to an individual formulation. Efficiency is determined by the position of each point relative to this frontier, with impact performance considered as the output parameter. Based on this analysis, the formulation containing 1.0 wt% diethanolamine was identified as the most efficient, as increasing the concentration to 1.5 wt% does not justify the additional input. Although the 0.5 wt%, 1.0 wt%, and 1.5 wt% formulations lie on the efficiency frontier. The increased variability at certain 1.0 wt%, and 1.5 wt% formulations lie on the efficiency frontier, the selection of 1.0 wt% is supported by an input-oriented criterion, as it minimizes diethanolamine content while maintaining equivalent impact performance.

These trends are reinforced by Table 2, which summarizes the effect of DEOA concentrations on the dominant chemical reactions, the resulting structure, and the relative efficiency. Concentrations between 2 wt% and 3 wt% excessively increased foam stiffness, compromising the ability for progressive deformation during impact. On the other hand, concentrations below 0.5 wt% did not provide enough hydroxyl groups to enhance NCO conversion and stabilize the cellular morphology, resulting in inconsistent mechanical responses.

The relationships presented in Table 2 are supported by literature reports on structure–property correlations in polyurethane foams [1,8,11,28].

Concentrations between 2 wt% and 3 wt% increased foam stiffness and compromised controlled deformation capacity, resulting in poorer energy dissipation and higher residual acceleration. This behavior is characteristic of excessively crosslinked foams, which absorb less energy through plastic deformation and tend to fracture more easily under severe impacts.

Conversely, concentrations below the efficiency threshold (0–0.5 wt%) do not provide sufficient reactive groups to improve cell uniformity and isocyanate group conversion, resulting in less compact foams with greater structural variability and inconsistent mechanical performance. Thus, DEOA acts as a property modifier, but its function is highly dependent on the optimal proportion.

The presented results are consistent with the literature [26,27,28,29,30], which indicates that DEOA, acting as a nucleophilic agent and potential chain extender, increases urethane bond density, reduces structural defects, and provides greater homogeneity to the foams. While this behavior had already been observed for flexible foams, this study demonstrates that it also applies to rigid polyurethane foams. The use of DEOA as a reactive additive therefore improves cellular structure efficiency up to a certain limit, beyond which excessive crosslinking causes the material to lose its elastic and plastic deformation capacity, two fundamental mechanisms for impact energy absorption.

Thus, the application of DEA in conjunction with DEA-based efficiency analysis proves to be a viable methodological tool to avoid misleading interpretations based solely on individual impact values. While classical analyses might suggest contradictory trends, DEA enables the integration of multiple parameters, revealing that the key objective is not to maximize or minimize a single value, but rather to optimize the relationship between formulation and overall performance.

DEA analysis proved to be essential for integrating multiple parameters and avoiding conclusions based on isolated data. Based on the FDH and VRS analyses, it was confirmed that the formulation containing 1% DEOA is the most efficient; therefore, only this foam was selected for complementary characterizations (SEM, FTIR, and TGA/DTG), as it represents the most relevant condition for subsequent characterization in terms of impact attenuation performance.

### 3.2. Ftir-Atr

**Figure 6:**
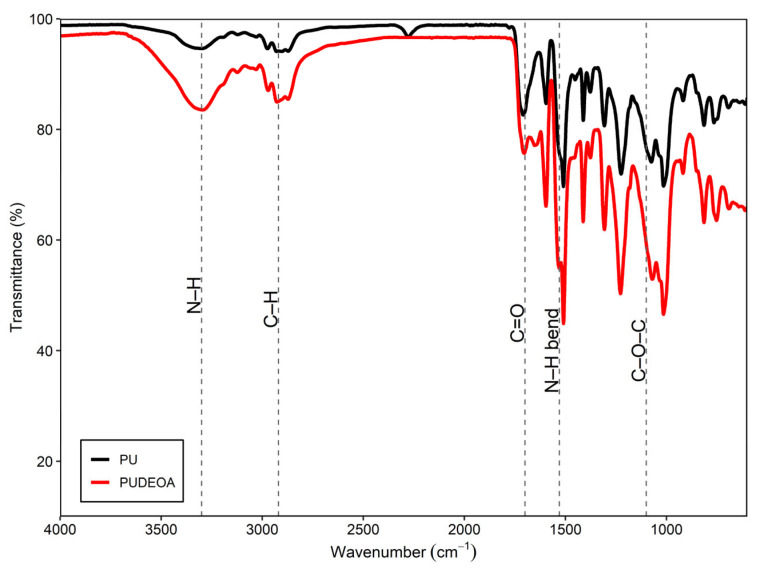
ATR-FTIR Polyurethane foams.

The ATR-FTIR spectra of polyurethane (PU) and polyurethane containing 1 wt% diethanolamine (PU-DEOA) are presented in Figure 6. FTIR analysis was performed to investigate the chemical modifications promoted by DEOA incorporation and to identify possible changes in the characteristic functional groups of the polyurethane network. Attention was given to the regions associated with N–H stretching, urethane carbonyl (C=O) vibrations, C–N and C–O–C bonds, as these bands provide valuable information regarding hydrogen bonding, urethane formation, and the overall organization of the polymer structure.

Overall, both spectra exhibit the characteristic bands of rigid polyurethane foams, including N–H stretching, C=O vibrations of the urethane group, and bands associated with soft-chain segments. However, important changes are observed when 1% DEOA is incorporated, reflecting structural modifications consistent with the literature [29,30,31,32] and with the mechanical effects detected in the impact tests.

The broad band between 3200 and 3500 cm^−1^ arises from N–H stretching vibrations and overlaps with O–H groups engaged in hydrogen bonding. The PU-DEOA sample exhibits higher intensity in this region, suggesting an increase in hydrogen-bonded structures, likely due to additional hydroxyl groups from DEOA.

The absorption band in the range of 1700–1730 cm^−1^ corresponds to the C=O stretching of urethane groups. A slight reduction in intensity is observed in the PU–DEOA spectrum, suggesting modifications in the chemical environment of carbonyl groups, possibly due to the formation of secondary urethane or urea-type structures.

In the region between 1500–1300 cm^−1^, associated with N–H bending and C–N stretching vibrations, the PU–DEOA foam exhibits increased band definition, indicating the incorporation of nitrogen-containing groups into the polymer network.

The region between 1200–1000 cm^−1^, related to C–O–C stretching vibrations, shows noticeable changes in the PU–DEOA sample, suggesting alterations in the polymer backbone organization and no residual isocyanate band (~2270 cm^−1^) was observed, indicating effective NCO conversion.

Overall, the spectral differences suggest that the addition of 1 wt% DEOA modifies the chemical structure of the polyurethane network, possibly increasing hydrogen bonding interactions and favoring a more homogeneous distribution of urethane linkages. These changes are consistent with the improved cellular morphology and impact attenuation performance observed for this formulation.

### 3.3. Apparent Density Analysis

The apparent density of the polyurethane foams was evaluated using cubic specimens with a volume of 27 cm^3^. The specimens were collected from different regions of the foam samples to consider possible density variations generated during the foaming process. The apparent density values obtained for the neat PU and PUDEOA foams are presented in Table 3.

The neat PU foam exhibited an average apparent density of 0.0679 ± 0.0054 g·cm^−3^, whereas the foam containing 1 wt% DEOA presented a higher apparent density of 0.1008 ± 0.025 g·cm^−3^. Therefore, the incorporation of DEOA resulted in an increase of approximately 48% in apparent density compared with the unmodified polyurethane foam.

This result is important because the 13.8% reduction in transmitted peak acceleration occurred together with an approximately 48% increase in apparent density. Since density strongly affects the compressive and impact behavior of polyurethane foams, the improvement should not be attributed exclusively to the chemical action of DEOA. Rather, it reflects a combined density, morphology, and network effect, in which higher apparent density contributes to load-bearing capacity, while cellular organization and network structure may favor stress distribution during impact.

The increase in apparent density indicates that DEOA promoted a more compact cellular structure, with a higher polymer fraction per unit volume. Diethanolamine acts as a reactive chain extender and crosslinking agent through its hydroxyl and secondary amine groups, accelerating network formation during foaming and modifying the balance between blowing and gelling reactions. As a consequence, cell stabilization occurs earlier, restricting excessive expansion and leading to foams with higher apparent density and improved structural integrity [29,30].

The addition of DEOA appears to modify this expansion behavior by promoting earlier network formation and improving cell stabilization during foam growth, which is consistent with the morphological differences observed in the SEM micrographs.

The higher standard deviation observed for the PU–DEOA formulation suggests a more pronounced density gradient along the foam thickness. This behavior is associated with the different expansion conditions experienced by the material during molding, including local variations in confinement, heat transfer, and cell growth kinetics. Such gradients are commonly observed in molded polyurethane foams due to the heterogeneous expansion process.

The density increase observed after DEOA incorporation is consistent with the morphological modifications identified by SEM analysis, which revealed changes in pore distribution and cellular organization. These results indicate that DEOA modifies not only the chemical structure of the polyurethane network but also the final cellular architecture of the foam.

Thus, the performance gain should be interpreted as a trade-off between increased apparent density and improved impact attenuation. Although the relative reduction in transmitted acceleration is smaller than the relative increase in density, the optimized PU–DEOA formulation remains relevant because it combines lower transmitted acceleration with improved cellular organization under identical testing conditions.

### 3.4. Morphological Analysis (SEM) of Polyurethane Foams with and Without Diethanolamine

The SEM analysis was used to provide both a qualitative comparison of the cellular morphology of the selected foams and a limited quantitative assessment of selected morphological features obtained from image analysis. Accordingly, the micrographs were first interpreted qualitatively to identify differences in cell organization, pore distribution, and regional heterogeneity, while ImageJ^®^ analysis was subsequently applied only to regions where image contrast and segmentation were sufficiently reliable. Therefore, the quantitative descriptors reported in this section should be interpreted as comparative and region-specific indicators, rather than as exhaustive morphological parameters for the entire foam structure.

**Figure 7:**
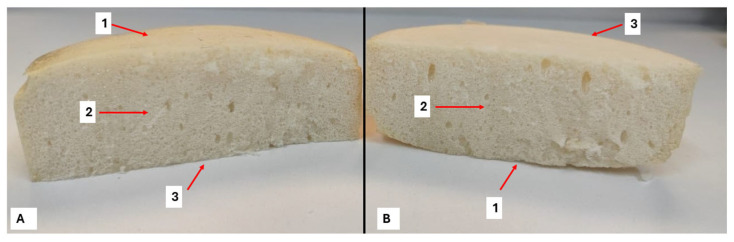
Representative images of the polyurethane foam showing the cross-sectional regions selected for characterization. (**A**) Foam in the direct orientation and (**B**) foam in the inverted orientation. In both cases, region 1 corresponds to the upper cross-sectional region, region 2 corresponds to the internal core region, and region 3 corresponds to the bottom cross-sectional region, which remained in direct contact with the mold during the foaming process.

A schematic representation of the regions selected for SEM analysis is shown in Figure 7. Micrographs were acquired from three distinct locations of the foam specimens, namely the upper surface, the internal core region, and the bottom region, to evaluate possible variations in cellular morphology along the foam structure.

**Figure 8:**
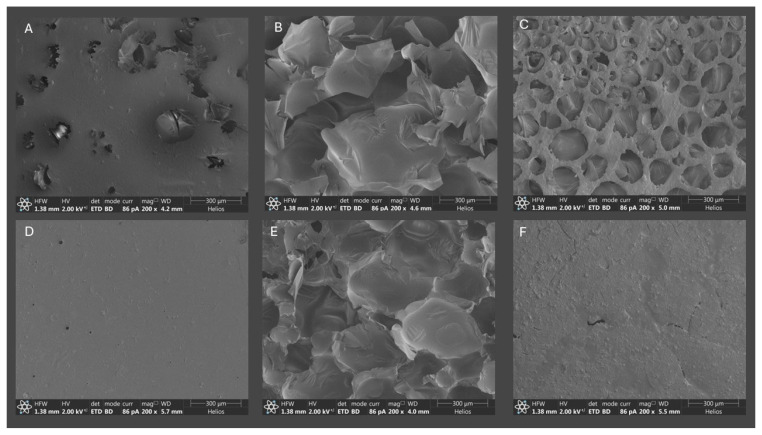
SEM micrographs obtained from different regions of the foam specimens: upper surface region (**A**,**D**), internal core region (**B**,**E**), and bottom region (**C**,**F**). Images A–C correspond to neat PU, whereas images D–F correspond to PU containing 1 wt% DEOA.

Figure 8 presents the SEM micrographs obtained for the neat polyurethane foam (PU) and the optimized formulation containing 1 wt% diethanolamine (PU–DEOA), selected according to the efficiency criterion obtained from the DEA analysis. The upper images (A–C) correspond to neat PU, whereas the lower images (D–F) represent the PU–DEOA formulation. The micrographs were obtained from the upper surface region (A, D), internal core region (B, E), and bottom region (C, F).

Figure 8 reveals the characteristic heterogeneous cellular morphology of polyurethane foams; however, significant differences are observed after DEOA incorporation. The neat PU foam exhibits greater morphological irregularity, with variations in cell size, non-uniform pore distribution, and regions with thicker cell walls. These characteristics indicate a less controlled cellular growth process during foam formation. In contrast, the PU–DEOA foam presents a more organized cellular structure, with improved pore distribution and reduced occurrence of highly irregular regions, suggesting that DEOA contributes to a more controlled cell formation and stabilization process.

To quantitatively evaluate the morphological modifications, the SEM images were analyzed using ImageJ^®^ software. The micrographs were calibrated using the scale bar, and the pore regions were separated from the polymer matrix by threshold processing. Particle analysis was subsequently performed using the Analyze Particles function to determine the number of pores, pore area fraction, and morphological parameters. For the upper surface region, the neat PU foam presented 685 detected pores, whereas the PU–DEOA foam exhibited 50 pores. The calculated pore area fraction was approximately 0.15% for PU and 2.0% for PU–DEOA, indicating that the incorporation of DEOA altered the cellular arrangement, resulting in fewer detected cavities with different geometric characteristics.

For the bottom mold surface, the morphological differences between formulations were more evident. The neat PU foam presented 546 detected pores and a pore area fraction of approximately 17.5%, while the PU–DEOA foam showed 97 pores and approximately 5.2% pore area fraction. These results demonstrate that DEOA significantly affected the cell development process, promoting a more compact cellular morphology with modified pore distribution.

The internal core region was not quantitatively analyzed due to limitations associated with image segmentation. In this region, the presence of very thin cell walls and insufficient contrast between the polymer matrix and cellular voids prevented reliable separation of pores during threshold processing. Consequently, quantitative analysis could generate inaccurate pore identification; therefore, this region was evaluated only qualitatively.

The differences observed between the upper and lower regions confirm the presence of a cellular gradient along with the foam thickness. This behavior is associated with the different conditions experienced during molding, including confinement effects, heat transfer, and local variations in expansion kinetics. The addition of DEOA modifies this expansion behavior consistent with the increase in apparent density previously discussed.

The morphological changes identified by SEM are also consistent with the improved impact attenuation performance observed for the PU–DEOA formulation. Although the addition of DEOA reduced the pore fraction in some regions, the impact results indicate that energy absorption is not controlled only by the total amount of porosity. Instead, the stability, distribution, and deformation capability of the cellular structure are key factors governing impact response. The improved impact attenuation is likely associated with the combined effects of increased apparent density, modified cellular morphology, and changes in the polyurethane network promoted by DEOA, which may favor progressive deformation and improved stress distribution during impact loading, contributing to the reduction in transmitted peak acceleration.

Overall, the SEM analysis indicates that 1 wt% DEOA modifies the cellular architecture of polyurethane foam, promoting a better balance between structural organization and mechanical response. These morphological changes support the improved impact performance obtained for the optimized PU–DEOA formulation.

### 3.5. Thermogravimetric Analysis

**Figure 9:**
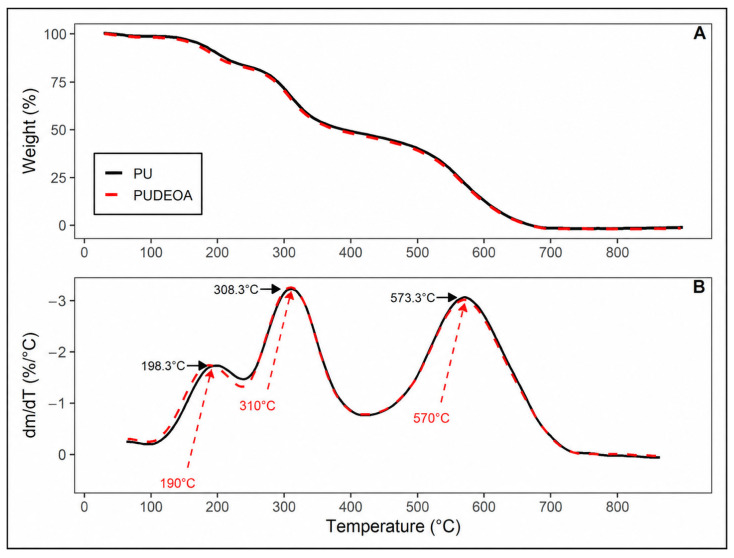
Thermogravimetric analysis (TGA) and derivative thermogravimetry (DTG) curves of polyurethane (PU) and polyurethane modified with diethanolamine (PUDEOA). (**A**) TGA curves showing the weight loss (%) as a function of temperature. (**B**) DTG curves highlighting the thermal degradation stages, with peak decomposition temperatures indicated for both samples. The results reveal a multi-step degradation behavior, with characteristic peaks associated with the breakdown of hard and soft segments of the polymer structure.

The thermogravimetric (TGA) and derivative thermogravimetric (DTG) curves of PU and PU–DEOA are presented in Figure 9. Both materials exhibit the typical multi-stage thermal degradation behavior of polyurethane foams.

An initial mass loss below 200 °C is observed for both samples and is attributed to the evaporation of residual moisture and low-molecular-weight species. The DTG curves show a first degradation peak at approximately 198 °C for PU (≈190 °C for PU–DEOA), which is associated with the dissociation of less stable urethane structures.

The main degradation stage occurs between 250 and 400 °C, with a prominent DTG peak at around 308 °C (≈310 °C for PU–DEOA), corresponding to the breakdown of urethane linkages and hard segments. This behavior is strongly supported by the FTIR spectra, which exhibit characteristic absorption bands of urethane groups, including N–H stretching vibrations around 3300 cm^−1^ and C=O stretching near 1700 cm^−1^. The cleavage of these bonds contributes significantly to the mass loss observed in this temperature range.

Additionally, the FTIR spectra show bands in the region of 1000–1200 cm^−1^, attributed to C–O–C stretching vibrations of the polyol segments. The degradation of these structures contributes to the subsequent mass loss at higher temperatures.

A second major degradation peak is observed at approximately 573 °C (≈570 °C for PU–DEOA), which is related to the decomposition of the remaining polymer backbone and carbonaceous structures. The presence of bands in the 1500–1600 cm^−1^ region suggests more thermally stable structures, which may contribute to delayed degradation at higher temperatures.

Comparatively, neat PU exhibits slightly higher onset temperatures (T_onset_), indicating marginally greater initial thermal stability. The incorporation of DEOA does not significantly alter the overall thermal stability; however, slight shifts in DTG peak temperatures suggest modifications in the degradation kinetics. These changes are consistent with the FTIR results, where variations in band intensity – particularly in the N–H and C=O regions – suggest differences in hydrogen bonding interactions and crosslink density. The presence of DEOA likely modifies the polymer network, influencing chain mobility and thermal decomposition behavior.

At higher temperatures, both samples exhibit similar degradation profiles, with negligible residual mass, indicating nearly complete decomposition of the organic material.

Table 4 summarizes the onset degradation temperatures (Tonset) and peak degradation temperatures (Tp) of the polyurethane foams. It is important to note that negative residual mass values were adjusted to zero, as they arise from baseline fluctuations inherent to TGA measurements.

Overall, the results indicate that the incorporation of 1 wt% DEOA slightly modifies the thermal degradation behavior without producing a practically significant improvement in thermal stability. These changes are therefore interpreted as complementary evidence of structural modification rather than as direct evidence of enhanced thermal resistance.

## 4. Conclusions

The results show that diethanolamine affects the structure, chemical behavior, and impact performance of rigid polyurethane foams, helping to clarify its role in this class of materials.

Apparent density was incorporated into the interpretation of the results, although the assessment was limited to the selected formulations and did not fully resolve possible spatial density gradients within the molded foams. Therefore, the observed impact performance should be interpreted together with the measured density values, cellular morphology, and chemical-structural evidence.

Accordingly, the 13.8% reduction in transmitted peak acceleration should be interpreted considering the approximately 48% increase in apparent density. Although densification likely contributed to the improved response, the optimized formulation remains relevant because the density increase was accompanied by morphological and network-related changes that support impact attenuation.

Higher concentrations (≥2%) appear to promote increased structural rigidity, likely associated with a higher degree of crosslinking and increased structural stiffness, reducing the capacity for progressive deformation and compromising energy dissipation. In contrast, concentrations below 0.5% resulted in foams with irregular morphology, partial NCO group conversion, and inferior mechanical performance.

Spectroscopic evidence revealed consistent changes with a more balanced formation of urethane structures. Concurrently, Scanning Electron Microscopy (SEM) morphological analysis indicated that the 1 wt% DEOA formulation achieved more homogeneous cells with visually thinner walls, which suppressed severe density gradients within the mold and favored the controlled dissipation of impact energy. Thermal behavior also corroborated the stability of the optimized formulation.

Within the scope of this study, the formulation containing 1 wt% DEOA showed the best overall performance and was identified as the most efficient among the tested conditions according to the adopted DEA criteria, while recognizing the measured density differences and the limited range of formulations evaluated. Based on these findings, future work should focus on developing rigid polyurethane composites reinforced with natural fibers to enhance impact performance and sustainability, while also incorporating systematic density measurements, density-normalized impact metrics, and, where appropriate, benchmarking against established impact-absorbing materials to provide a more comprehensive assessment of formulation–structure–performance relationships.

## Figures and Tables

**Table 1:** Mean transmitted peak acceleration for polyurethane foams containing different DEOA concentrations.

DEOA (wt %)	Mean Transmitted Peak Acceleration (g)
0.0	72.88 ± 2.15
0.5	64.14 ± 1.87
1.0	62.79 ± 1.42
1.5	63.40 ± 2.01
2.0	67.89 ± 2.56
2.5	85.52 ± 3.74
3.0	115.27 ± 4.98

**Table 2:** Effect of DEA Content on Chemical Structure and Efficiency.

DEA (%)	Dominant Chemical Reaction	Formed Structure	Impact on Efficiency (VRS)
0–1.0	Balanced formation of urea/urethane linkages	More favorable cellular structure	High efficiency (on frontier)
>1.0	Increased formation of urea/urethane linkages and possible over-crosslinking	Rigid/excessive structure	Inefficiency (points above the frontier)

**Table 3:** Apparent density values were calculated from cubic specimens collected from different regions of the foam samples.

Sample	Specimen (cm^3^)	Apparent Density (g·cm^−3^)
PU	9	0.0679 ± 0.0054
PUDEOA	9	0.1008 ± 0.0250

**Table 4:** Onset (Tonset) and peak degradation temperatures (Tp) of polyurethane foams under N_2_ atmosphere (10 °C·min^−1^).

Sample	Tonset_1_ (°C)	Tonset_2_ (°C)	Tonset_3_ (°C)	Tp (°C)	Residual Mass (%)
PU	166	281	534	308	0.00
PU-DEOA	156	279	523	310	0.00

## Data Availability

The original contributions presented in this study are included in the article. Further inquiries can be directed to the corresponding author.

## Abbreviations

The following abbreviations are used in this manuscript:

ABNT	Brazilian Association of Technical Standards
ASTM	American Society for Testing and Materials
ATR-FTIR	Attenuated Total Reflectance–Fourier Transform Infrared Spectroscopy
DEA	Data Envelopment Analysis
DEOA	Diethanolamine
DTG	Derivative Thermogravimetry
EPS	Expanded Polystyrene
FDH	Free Disposal Hull
INT	National Institute of Technology
LAENP	Product Testing Laboratory *(Laboratório de Ensaio de Produtos)*
NBR	Brazilian Standard
PPE	Personal Protective Equipment
PU	Polyurethane
SEM	Scanning Electron Microscopy
TG	Thermogravimetry
VRS	Variable Returns to Scale
